# The Hospital Nursing Supplement

**Published:** 1894-09-29

**Authors:** 


"The Hospital" Nursing Mirror.

Being the Extra Nursing Supplement of "The Hospital" Newspaper.

[Contributions for this Supplement should be addressed to the Editor, The Hospital, 428, Strand, London, W.C., and should have the word "Nursing" plainly written in left-hand top corner of the envelope.]

### News from the Nursing World.

### OUR CHRISTMAS COMPETITIONS.

It is time to begin to think seriously of the needlework competition, in which our readers always take such a kindly interest. We hope that already many of them are commencing some of those delightfully warm garments which form such valuable gifts for the sick poor in hospital on Christmas Day. To leave comfortable wards, nourishing food, and adequate blankets for barren, poverty-stricken homes is a sad enough change at any time, but worst of all in cold January weather. New flannel is an unattainable luxury in many a family where the bread-winner's illness has disastrously curtailed even the necessaries of life. Therefore we plead boldly for kindly co-operation in securing a goodly supply of suitable garments for those to whose complete convalescence they are absolutely essential.

### ROYAL NATIONAL PENSION FUND.

After this week, the address of the Royal National Pension Fund for Nurses will be 28, Finsbury Pavement, London. The new offices are easily accessible by trams, trains, and omnibuses from all parts of the town and country.

### WINTER WORK.

A great variety of instruction is provided for the London Young Women's Christian Association this winter. The members are taught gymnastics, at moderate fees, at the Central and Finsbury Institutes. Classes are also formed for the study of hygiene cookery, nursing, dressmaking, bookkeeping, and French. All particulars can be obtained from the Secretary, 16a, Old Cavendish Street, London.

### ATTACK UPON A MATRON.

It is reported that the matron of the Stanley Hospital, at Holyhead, has received very serious injuries at the hands of a patient. Becoming suddenly insane, he is said to have rushed after the unfortunate lady and attacked her in a most violent manner before he was secured. The condition of the matron is extremely serious.

### IN CASH OR IN KIND?

Ambulance classes appear to be sometimes held under the auspices of the Technical Education Branch of the County Council, and in a district where this custom prevails a resident medical officer offered to give the course of lectures for five guineas. He was annoyed by a report that a doctor from a town five miles off had volunteered to do the work for nothing. But voluntary lecturers often accidentally receive substantial rewards which far exceed five guineas in value. Each year "presentations" are of frequent occurrence, and only the other day it was recorded that a silver tankard, cigarette-case, inkstand, and silver knives formed the "token" of the esteem in which a medical lecturer was held by his classes. Instances of women lecturers getting payment in this fashion are unusual; probably they prefer "fair wages for fair work," and see obvious advantages in making their own purchases with their own earnings.

### SICK, BUT NOT CERTIFIED.

"I do not sign my name, as I do not wish in any way at present to give a clue to the identity in this sad case," writes "Amicus" in a daily paper last week. The heading, "Workhouse Brutality," is sufficiently alarming for the reader to experience relief on finding that the letter relates neither to cruelty nor neglect of paupers, attention being simply claimed for a respectable young man, who "needed to be placed under medical control and care," and pending the doctor's verdict on his mental condition, his friends placed him in a metropolitan workhouse infirmary. The "brutality" complained of, consisted chiefly in his having been dressed like the other inmates. "Amicus" considers that he ought not to have been compelled to submit to this, although, as he was temporarily a patient, it seems natural that he should be treated in this respect like the other sick poor. His request to "Amicus" to visit him, showed that he was allowed to invite friends. The wearing of "a pauper's livery" and the absence of books, appear in fact, his only grievances. Both could only be remedied for the whole community, and a supply of books and newspapers would be welcomed at most workhouses if the public would send them. The "pauper's livery" is hated by all the decent poor and valued by the worthless because it secures alms in the streets; its abolition has been often discussed, and until this takes place such cases as that described by "Amicus" must often occur, for his friend is not the first honest man who has felt bitterly the degradation of the outward badge of poverty!

### RECOMMENDATIONS.

The word "recommendation" is defined in a dictionary as "that which procures a kind or favourable reception. But the recommendations made by the Local Government Board are not invariably thus received, especially when they refer to the subject of trained nursing for paupers. In one place the advice of the medical inspector was recently set at naught because the master of the workhouse and a guardian or two chose to consider the existing condition of things satisfactory. In other cases half-measures take the place of suggested wholesale reforms. Rarely, indeed, do principles, or the fear of public opinion, secure the favourable reception which the dictionary asserts to be the due of recommendations in general.

### DISTRICT NURSING IN THE ISLE OF WIGHT.

The annual meeting of the Newport (Isle of Wight) District Nursing Society was held in the Guildhall last week, and was very well attended. The Mayor presided, and the committee offered their fifth annual report, which shows that Nurse Butcher's services have been much in request during the year. New subscribers are urgently needed to replace those lost through death or other change.

### EQUAL ADVANTAGES.

In arranging their classes at the Mechanics' Institute, Coniston, for the coming winter, it was decided that a course of instruction on ambulance and sick nursing should be given to the male and female students respectively. This example might well be followed in other places, for many men prove apt pupils, and feel decidedly more useful members of society when they can act wisely and skilfully in emergencies.

### AN AMBULANCE REQUIRED.

The Committee of the Stalybridge Sick Nursing Association are desirous of raising a fund for the purchase of a tricycle ambulance for the conveyance of cases of accident to the District Infirmary. Doubtless the idea will commend itself to many liberal people as soon as they reflect upon the unnecessary amount of suffering inflicted frequently during the transit of the injured by unsuitable conveyance.

### NURSES IN IRELAND.

The introduction of trained nurses to the sick poor in their own homes has been an unqualified success in Ireland. Additions to the number of workers are frequently recorded, and a recent meeting at Enniscorthy proved the cordiality with which members of all denominations unite in promoting the common good. This gathering was held in the Athenæum, and was well attended by members of the District Nursing Association, much pleasant evidence being volunteered respecting the valuable work accomplished by the nurse. The Superintendent of the Irish Branch of the Queen's Jubilee Institute, Miss Dunne, gave an interesting description of the progress of district nursing in other parts; "there were seventeen districts with nurses already working, and in no place had the nurse been allowed to go away after her services were once experienced." The other speeches were also excellent, and it is evidently the intention in the Enniscorthy district to set a good example to the neighbours. Nurse Fielder is to be congratulated on the keen appreciation of her work which exists amongst the patients and their friends.

### A LIBERAL FRENCHMAN.

M. Henri Schneider has founded a new Hôtel Dieu, at Creuzot, which he has presented to the town; it was opened on the 18th inst., and contains, it is said, 150 beds. The Benediction at the ceremony was invoked by Monsignor Pérraud, member of the Academy, and Bishop of Autun.

### FOR CANCER PATIENTS.

A new pavilion is in course of erection in the grounds belonging to the Empress Elizabeth Hospital at Rudolfsheim. It is to contain a number of small wards for the use of female cancer cases, and all the plans for the new building show great consideration for the comfort of the patients. The cost of the pavilion will be defrayed out of the "Bettina Fund," and is to be completed in the course of next year and occupied soon afterwards. The "Bettina Fund," amounting to £50,000, is a gift from Baron Albert de Rothschild to the hospitals of Vienna.

### RUSSIAN PROGRESS.

If certain preliminaries can be arranged, Russian women have every prospect of being able to secure for themselves a full medical education in St. Petersburg. Two-thirds of the funds needed to found a medical institute for women are, indeed, already promised. Students must be between the ages of eighteen and thirty-five, and the course of instruction will take six years, the last two being devoted to "practical work in hospitals devoted to obstetrics and the diseases of women and children."

### OUR AMERICAN SISTERS.

The American Society of Superintendents of Training Schools for Nurses admits as members only "graduates in good and regular standing from training schools connected with incorporated general hospitals giving not less than a two years' course of instruction." Only trained superintendents of good standing are therefore eligible, assistant superintendents being admissible as associate members if they be "regularly appointed assistant superintendents of training schools for nurses that are regarded to be properly such by the Council; and they are eligible for such membership only during the time they are holding such appointments."

### THE EMPRESS OF JAPAN.

The Japanese Red Cross Society, which has for its chief patroness the Empress of that country, is at present busily engaged in preparing lint and in making bandages. The ladies of the Court are assisting her Majesty to get ready supplies, which are for the use of Japanese and Chinese.

### SHORT ITEMS.

A conference is soon to be held in Dublin of the medical officers of Irish workhouses, from which it is hoped that some definite action will result, as the condition of the sick poor in Ireland leaves much to be desired.—£11 was added to the Parish Nurse Fund by an entertainment lately given in the Abbey Room, Somersham.—Lady Mackenzie of Gairloch recently organised a fancy fair with the aid of some of her friends, on behalf of the Rosskeen Branch of the Western Ross Nursing Association. The Countess of Cromartie, in formally opening the fair, explained that the object of the association was the establishment of a trained nurse for the sick poor in their own homes.—A private nurse at Cardiff had a rib accidentally broken by a blow received in the street from a piece of soap thrown by a boy from an alley by which she was passing.—Miss Curtis received a handsome brass standard lamp, with a suitable inscription, on her resignation of her post at the Home for Nurses, Fitzwilliam Street, Cambridge.—The Lord Mayor of London is to lay the foundation-stone of the New Caxton Convalescent Home at Limpsfield, Surrey, on Saturday afternoon, October 6th.—The work of the Aberdeen District Nursing Association is increasingly valued, but the financial support accorded is, so far, inadequate.—530 visits were paid by the West Hartlepool District Nurses last month.—Nurse Hinton has become a district nurse.

### Diet in Disease.

By Mrs. Ernest Hart.

Series II.

### IV.—TYPHOID FEVER.

**Typhoid fever is caused** by the presence in the intestines of a minute bacillus which is most commonly introduced into the body by the means of polluted water or milk, and, as some suppose, by aerial transport in sewer gas, &c. It finds its proper soil or nidus for vigorous life and multiplication in the solitary glands and the Peyer's patches of the small intestine, as well as in the mesenteric glands. The solitary glands of the small intestine are about the size of a millet seed, and are found scattered over every part of the mucous membrane of the ileum. They are simple in structure, being composed merely of dense net-like tissue, the meshes of which are closely packed with lymph corpuscules. They are pervaded by fine capillaries and surrounded by a rich plexus of lymphatic vessels. The nodules bulge towards the interior of the gut and their bases are situated in the submucous tissue. Their upper surfaces are free from villi. Peyer's patches are simply collections of solitary glands. They are oblong in shape, and are about an inch in width and from half an inch to two or even four inches in length; they are found placed lengthways opposite the side of the intestine which is attached to the mesentery.

**The Morbid Changes in the Intestine.**—When a person is infected with typhoid by swallowing the bacilli typhi with the water or food, the microbes make their way to the Peyer's patches and solitary glands, where they find their proper soil. They here multiply, and by their irritating presence, as well as by the poisonous substance they excrete in the process of living, cause inflamamtion of the closed follicles. The glands swell up and become solid, ulceration follows, and the slough is finally separated and thrown off. The walls of the glands are succulent and vascular, and considerable hæmorrhage may be caused by sloughing. The ulceration may, moreover, extend downwards through the muscular coat, even into the serous coat, and result in perforation of the intestine. If the process is gradual the inflammation generally extends to the peritoneum; fibrinous exudations are thrown out, and adhesions take place between the peritoneum and the thinned walls of the intestine which prevent perforation and the escape of the contents of the intestine into the abdominal cavity. The ulceration may be so severe and rapid as to cut off the blood supply of the serous coat, in which case the latter undergoes necrosis, perforation takes place, the contents of the intestine pass into the peritoneal cavity, and fatal peritonitis ensues.

**Periods of the Illness Corresponding to Morbid Processes.**—Inflammation of the follicles is contemporaneous with the first symptoms of illness. It reaches its culminating point about the tenth day. If the case is slight, resolution or the absorption of the products of inflammation then takes place slowly; or in severe cases the follicles ulcerate. The sloughs separate during the third week of the illness, and the process may not be completed until the fourth week. Cicatrisation of the raw surfaces of Peyer's patches begins about the end of the third week, and takes about two weeks to complete. Indiscretions of diet may, however, again set up inflammation, and Dr. Bristowe declares that the liability to perforation continues for from two to three months after the commencement of the illness.

**Physiological Rest the Rational Treatment.**—Knowledge of the processes of inflammation going on in the intestines of a typhoid patient gives the indication for treatment. This is physiological rest. Let the bowel remain as immobile as possible, so that cicatrisation of the raw, ulcerated, and bleeding patches inside it may heal, and let no impetus be given towards perforation by undigested particles of food setting up violent peristaltic action. The rest must be also muscular as well as physiological. A sudden movement of the patient may rupture the friable adhesions between the ulcerated intestine and the peritoneum, and the dreaded perforation into the peritoneal cavity may consequently take place. When the inflammatory process accompanied by fever is over, and appetite returns after the third week, the condition of physiological rest of the intestine must still be maintained as a leading principle of the diet. The slough is probably thrown off, but the raw surface is not yet cicatrised, and until it has done so the patient is not safe from the fatal accident of perforation, and in feeding him this is the first consideration.

**Typhoid Patients Sometimes Killed by their Friends' Kindness.**—Every hospital student can recall cases in which a promising typhoid case recovering from an acute attack has suffered a sudden relapse with, perhaps, perforation and death. On inquiry it is often found that the bad symptoms set in after "visiting day," and that kind friends sympathising with the patient's desire for solid food and his distaste for the rigid regime so long enforced have surreptitiously brought him plum-cake, fruit, or bread and jam, &c., which have been secretly eaten at the cost of a relapse, and perhaps even of his life. It is only necessary to study the pathological conditions described, and to appreciate the fact that the intestine is in typhoid ulcerated and raw, and its walls dangerously thinned at certain points in order to recognise the importance of these precautions.

**The Cardinal Principles of Dietetic Treatment,** which are as follows: (1) The maintenance of the strength of the patient; (2) to give the intestine the physiological rest necessary, in order to avoid the accident of perforation, and to favour repair and cicatrisation. In all acute fevers the metabolism of the tissues is abnormally active, and the waste great. The products of combustion are consequently present in abundance in the blood. Appetite is abolished, and stomachal digestion is suspended. Our object must, therefore, be to give the patient foods that are—(1) easily digested or rather absorbed; (2) that will not increase the amount of urates in the blood; (3) that will diminish the abnormal waste of the tissues. Hence the reason for the use of beef teas, jellies, arrowroot gruel, and milk in acute fevers. In typhoid it must, moreover, always be borne in mind that the food given must be in the form of bland fluids very easily digested.

**Milk.**—This is the aliment that suggests itself as the most appropriate for typhoid patients. It is a complete food, and on it alone a patient can subsist for an indefinite time. As a rule it is well borne, and in such cases it should be taken in small quantities at frequent intervals. It must, however, be borne in mind that though, out of the body, milk is a fluid, it is curdled at once in the stomach by the action of the gastric acids, and that consequently it may pass as a firm solid through the whole length of the intestine, and set up irritation, leading to injury. It is of the utmost importance that in typhoid the evacuations of the patient should be carefully watched. If the milk is excreted in the form of curds, this is an indication that the patient cannot digest pure milk. It must then be given diluted. Equal parts of milk and Vichy or Vals water, or one part of milk to two of Apollinaris or soda water, may be administered, or ten grains of bi-carbonate of soda and the same quantity of common salt may be added to every pint of milk and water in equal parts. Milk thus diluted and mixed with alkali can often be absorbed when pure undiluted milk would not be digested. (Yeo).

**Asses milk**, which is precipitated in the stomach as an extremely fine curd, and is consequently the more easily digested, may be substituted for cow's milk.

**Whey** may be usefully given where milk is ill-digested. It is made by boiling a pint of milk with a tea spoonful or two of lemon juice or rennet, straining through muslin, and squeezing all the fluid from the curd. Care should be taken to break up the curd with the fingers while pressing, for then much of the fat and some of the finely divided casein of the milk will pass into the whey and make it more nutritious.

**Eggs.**—These should not be cooked, but the yolks beaten up raw with boiling water or with hot broth.

**Beef-tea.**—The usefulness of beef-tea is universally acknowledged in acute fevers. It is valuable in many different ways it contains gelatine, which, as already described, prevents waste of the albuminous tissues: soluble salts, which compensate for the extravagant loss of these in fever; and certain stimulating substances dissolved out from the beef. Mutton and chicken broths and clear soups are useful, as well as beef-tea essence, which should not, however, be given in too concentrated a form. In preparing clear soups and broths vegetable juices should be added in order to supply the body with the salts and acids contained in vegetables, particularly needed in fever. It is important, however, to exclude the indigestible vegetable fibres from the soup. The vegetables used should, together with the aromatic herbs, be cut up fine and placed in a muslin bag and boiled. The juices should then be pressed into the soup or broth.

**Raw meat pulp** may be given in prolonged cases of typhoid. Thin oatmeal or barley gruel, carefully strained from all gritty and irritating particles, and flavoured with sugar and lemon peel, is one of the best of the farinaceous foods. The gruel can also be mixed with milk, beef-tea, or meat essences, and thus a useful composite food is produced.

**Water** is absolutely necessary for the fever patient to allay his consuming thirst. Barley water, toast water, pure iced water, soda and effervescing waters may be given, changing one for another as the patient tires of each.

**Alcohol** in any form is as a rule forbidden, and should only be given under medical advice.

**Diet During Convalescence.**—This is often extremely difficult to arrange. As soon as the temperature falls to normal, the appetite of the patient often becomes voracious. His piteous demands for food are almost irresistible, but they must, notwithstanding, be firmly resisted, for it must be remembered that the intestine is still undergoing the process of ulceration and repair, and that solid food might set up fresh inflammation and cause fatal perforation. It is not safe to give any solid food till the temperature has been normal for at least eight days, and in severe cases for a longer period. The return to solid food should be very gradual. The beef-tea or soup can first be taken with the addition of fine bread crumbs; custards and jellies may then be added; eggs are admissible lightly poached or beaten up in broth, also oysters and boiled fish. Sandwiches made of pounded chicken between thin squares of bread may next be attempted, and if no bad consequences result a slice from the breast of boiled chicken may be eaten; but not until all danger is completely past may the patient again enjoy his mutton chop and rump steak.

**Dietetic Precautions for the Prevention of Typhoid.**—It must never be forgotten that typhoid fever is a filth disease, and that its most frequent cause is the pollution of drinking water by infiltration from drains or cesspools containing the specific poison of typhoid. To ensure protection against typhoid, it is first of all necessary to make sure of a perfectly pure drinking water; but as this is difficult if not impossible, under all circumstances, the drinking water should be always boiled. This is a precaution which should be invariably taken in all those countries where the laws of sanitation are not observed, as in Italy, Spain, and the East. A necessary part of the traveller's luggage is a small Etna, by which he can boil in his bed-room all the water he intends to drink. Milk, of the source of which we are not sure, should always be boiled before given children to drink. If these simple precautions were observed the cases of typhoid fever which not infrequently occur after a visit to the Continent would often be prevented.

### Notes from Germany.

Prussia alone can boast of possessing no less than 125 mineral springs. Thirty-four of these are to be found in the province of Schleswig-Holstein, and since the year 1870 the number of visitors to these health resorts has been increasing to an enormous extent. The visitors recorded in 1870, for instance, amounted to 99,009, and in 1875 they reached a total of 183,794, and in 1890 401,499. Within the last twenty years the increase of visitors to seaside resorts has also been equally perceptible, the number last year having been 119,465, against 5,309 in 1873.

Wiesbaden contains twenty-four springs, and is apparently the favourite resort, receiving in 1890 102,028 persons. In the same year 12,920 visitors were registered at Homburg, but the latter has become such a fashionable place, it is hard to distinguish between those who go there in search of amusement and those who go to look for health.

Of the visitors who take advantage of German baths, by far the greater number come from Great Britain; next in proportion come Russians, Americans, and Austrians.

A new and simple means of ventilation which is especially adapted for use in sick rooms emanates from Munich. It consists of an oblong piece of metal which projects from the stove pipe into which it is fixed. The former contains a smaller piece of metal which forms a door, and which, by means of a regulator, can be opened and closed as required. When kindling a fire the apparatus must be closed to prevent the smoke penetrating into the room. Rooms heated by stoves are apt to get too warm, and by means of this apparatus an even temperature can easily be maintained. In summer it proves equally useful as a means of ventilation in the cooler hours of the evening and during the night. For a ward it would, of course, be wholly insufficient. It is estimated that in the space of an hour a room may be thoroughly ventilated and freed of all foul air by this apparatus.

Only specially shaped bottles are used for poisonous drugs, lotions, liniments, &c., so that disastrous mistakes in administering medicines rarely occur in Germany. A law has been in force for some years past dealing with this matter. Chemists here do not possess the right of prescribing in any way, and it is difficult to procure the simplest drug without a doctor's prescription.

An interesting book containing numerous illustrations and entitled "Medical Science in the United States" has recently been published by G. Thienic, Leipzig. Dr. Med. S. Placzek is the author of it. He collected his information last year when on a visit to the States, and nothing seems to have escaped his notice. He gives a full description of the arrangements and working of several hospitals and asylums, and his book is well worth perusing by those interested in these matters.

The Evangelical cemeteries were as usual visited by thousands on Midsummer Day. No one who has never visited the cemeteries here can imagine their beauty. Everybody, without exception, poor and rich alike, goes laden with flowers to lay on the graves of their dear ones. Scarcely a mound is bare; even the last resting place of the French soldiers who fell at the battle of Leipzig in the world-forgotten graveyard of St. Johannes are decorated. Their generous adversaries, the Germans, pay their tribute of respect to bravery, and the graves were pointed reverently, "See, these are their graves; they fought bravely, and we do not forget them."

The difference between these churchyards and English cemeteries, too, is very great. Many here are beautiful gardens, the scent of flowers fills the air; trees are everywhere. Nothing can be heard except the twittering of the birds overhead and the humming of the bees, and involuntarily come the words, "It is indeed God's acre."

At sunrise bands of students wend their way to the graves of their deceased comrades and professors, and here by the graveside they sing the student songs they sung together of old, and lay their wreaths on the mounds before them.

☞ For New Laundry Hostel at Guy's, Nursing in Holland, Everybody's Opinion, &c., see p. ccxlix. et seq.

### The New Laundry Hostel, Guy's Hospital.

At the end of last June this new home for the laundry staff at Guy's Hospital was opened by the Bishop of Rochester, and is now in full working order. Before shortly describing the building itself, a few words of explanation as to the scope and general intention of this scheme for the housing of the laundry hands within the hospital precincts may be given, it being a departure which is quite new in the history of hospitals.

The laundry of an institution of the size of Guy's Hospital is an important department indeed, and its efficient management a matter of no small anxiety to those responsible for it. At Guy's, as at other large hospitals, the yearly list of "missing" linen, blankets, sheets, &c., has assumed very large proportions, amounting in money value to some £100 per annum, and the complaints from sisters and nurses of the unavoidable thefts through dishonest washerwomen and of the inefficient washing have been many and great. The authorities at Guy's, therefore, came to the conclusion that something to remedy the existing evils must be done, and the provision of a home for resident laundresses within the hospital grounds, under the supervision of a competent mistress was the plan decided upon, a plan which has been carried out with a thoroughness in every detail such as reflects the greatest credit on its promoters.

But the great influence for good that such an institution may be justly expected to have, is, after all, the most important aspect of the scheme. The treasurer, in the account of the home and its reasons for establishment, reminds those who know something of hospital work of the great difficulty experienced in finding "suitable employment for young women who, more often from ignorance than deliberate intention, have fallen out of the way of right living." When such girls, having profited by their stay in the wards, and anxious to lead better lives, are discharged from the hospital, too often the finding of honest work is almost impossible. "Upon the ground that such cases are peculiarly fit subjects for the charity of the Samaritan Fund . . . . the treasurer gained the consent of the trustees of the Spicer bequest to the proposal to use some of the money in their hands upon the purchase of th hostel, interest upon the capital thus expended being guaranteed by the hospital, and such interest being applied in accordance with the terms of the legacy, for the relief of convalescent patients on their discharge."

At the right moment a building, originally stables, with coachman's rooms, &c., became available for purchase, and this was accordingly effected at a cost of £2,100, a further £1,500 being spent in converting the place into the charming home it now is. The position, immediately outside the hospital boundary—within, in fact, on one side—could hardly have been more convenient. The principal entrance is in Old King's Head Yard, a second door giving upon the hospital grounds, quite near the laundry itself.

Entering from the hospital side, the ground floor is found to contain a large sitting or recreation-room, the mistress's sitting-room, and the heating chamber. (The latter we have already noticed in The Hospital for August 11th, p. 399.) On the first floor is the dining-room, with a "serving-room," lavatory and bath-room, and a dormitory having five cubicles, overlooked from the mistress's bed-room. On the next floor is a large dormitory, with thirteen cubicles, the head laundress's bed-room, lavatory, and bath-room. The top floor contains the kitchen, scullery, larder, &c., and two or three small bed-rooms for use when required. Of lifts there are two, one for taking coals, &c., up to the kitchen, and another between dining-room and kitchen.

Nothing can exceed the daintiness of the cubicles, each provided with a "combination" wardrobe and chest of drawers, chair, &c., the warbrobe, especially designed by Dr. Perry, and screened off from the central aisle by pretty curtains of a washing materials. Each dormitory is provided with a plentiful supply of tip-up wash-hand basins with hot and cold water, at the end of the long room, and there are baths on each floor. The polished floors, dainty bed-spreads, and the many little comforts give a most attractive appearance.

Dr. Perry has taken the most active interest in every detail, and the result is what might be expected. A keen interest in the enterprise has been taken by all concerned, and a talk with Miss Evans, the late dormitory matron in the hospital, now the first mistress of the laundry hostel, shows how entirely her heart is in the undertaking.

It is confidently hoped that the scheme may widen out yet, and the hostel become of the greatest use in conjunction with the many other charities working for the rescue of fellow creatures in the great city. As a charity, therefore, as well as an important benefit to Guy's Hospital, this new development will command ready sympathy and good wishes on all sides, and its success is looked for with interest.

### Everybody's Opinion.

[Correspondence on all subjects is invited, but we cannot in any way be responsible for the opinions expressed by our correspondents. No communications can be entertained if the name and address of the correspondent is not given, or unless one side of the paper only be written on.]

### THE MEDICAL STAFF CORPS AND MALE NURSING.

"An Army Sister" writes: The statement that there is no training school for male nurses in England appears frequently in your valuable paper. May I call attention to the Medical Staff Corps? The men have to be soldiers as well as nurses, and have many hardships; but, given the desire to learn, no wider field of experience can be found than in a military hospital. To become a soldier involves a certain loss of social status, and becoming a nurse formerly did the same. Food and accommodation are somewhat rough in the army, and so they were in civil hospitals when trained nursing was in its infancy. Enlistment is binding, but so is an agreement with a training school. Some men will never make nurses, but many soldiers equal in training and experience any staff nurse in a metropolitan hospital. The weak point in the training of the Medical Staff Corps is that the men are not obliged to be for a definite time in a sister's ward, or even under a trained orderly or male nurse who has himself been in a sister's ward. Trained nursing can only be taught by a trained nurse, though the surgeons are most painstaking very often. There are many details in nursing which are quite out of the range of a medical officer's experience. He has gained knowledge in other ways. Nursing is undoubtedly a woman's work, but men learn to become as good and unselfish nurses as any women.

### NURSING IN PARIS.

A "Levick Nurse" writes: May I ask you to insert in The Hospital the following particulars as a warning to nurses who may be tempted to join a nursing institution in Paris called the "Levick" Institution. This institution was under the patronage of the Marchioness of Dufferin and Ava, but a year ago this lady withdrew her name, though this fact was not publicly known, as the circulars and rule forms of the institution were not altered. In July, 1893, I joined this place from a hospital where I had been a nurse for four years, and although the management was in many ways odd, to say the least of it, I personally had no reason to complain until last April, when I found that financial difficulties menaced the institution, and the salaries of the nurses were not to be obtained. On July 14th, 1894, I was interviewed by Mr. Levick, who was at the head of the institution with a matron during his mother's absence, and was informed that I and the other nurses must leave at once, as the institution was bankrupt and everything might be seized at any moment. We naturally requested the money due to us—in my own case £12 14s.—and were jauntily told that there was no money to pay us with, and that we must go at once. This after three days we were forced to do, as no food was provided for us at the institution, and as we three nurses were reduced to our last penny—in fact, were forced to borrow of friends—the passage to England was paid for us from the British Charitable Fund. I need not enter into the discomforts to which we were subjected, but as I have since learned that the Levick Institution is still open, in spite of their assurances to us that they could not continue it, I thought it only right that these facts should be made known to other nurses. Besides myself, there are several other nurses who have been in the institution during the last eighteen months to whom money is owing.

[We have ascertained that Lady Dufferin removed her name from this institution a year ago.—Ed. The Hospital.]

### NURSES' CLUB.

Nurse Winifred writes: I am anxious to know if the nurses of Liverpool would be willing to form a good club on the same principles as the one in Buckingham Street, London? They would then see the different medical journals and might have a library. A small annual subscription would meet the general expenses. I feel sure that a club would be a great boon to nurses who could go there and study quietly when not otherwise engaged, and more especially the nurses who are working on their own account.

### Notes and Queries.

### Queries.

(147) *Slander.*—Will you kindly tell me whether there is a fund or society for helping a nurse in an action for slander?—*H. M.*

(148) *Training.*—At what age can I enter a children's hospital?—*A Would-be Nurse.*

(149) *San Remo.*—Can you tell me whether it is too late for me to get engaged as one of the private nurses at San Remo this season?—*Trained Nurse.*

(150) *Sunny South.*—To whom should application be made for private nursing in South of France or Italy?—*E. A. S.*

(151) *Student.*—Will you kindly give me some information on certain points which seem to be contradictory in text books on digestion?

(152) *Stewardess.*—Where should I apply for a post?—*E. H.*

### Answers.

(147) *Slander (H. M.).*—We have never heard of one.

(148) *Training (A Would-be Nurse).*—Get "How to Become a Nurse," by Honnor Morten, published by "Scientific Press," 428, Strand.

(149) *San Remo (Trained Nurse).*—Miss Bryant, 19, Vittorio Emanuele, San Remo, has already engaged her nurses for the coming season. You might apply in January, if there is much sickness, as additional nurses are sometimes urgently needed in the Riviera after Christmas.

(150) *Sunny South (E.A.S.).*—See "Nursing" in Burdett's "Hospital Annual," which is published by "Scientific Press," 428, Strand.

(151) *Student.*—If you wi l devote more careful study to the subject of food and digestion in a recognised text-book, such as Michael Foster's or Landois and Stirling's "Physiology," you will see that there is no such contradiction between the functions of the blood-vessels and those of the lacteals as you suppose. Foods may certainly be divided into flesh formers, heat producers, bone formers, &c., as before, because inasmuch as flesh is formed, heat is produced, and bones are built up, they can only be built up and produced out of the raw materials, the different foods which are taken into the body. Whether physiologists are always strictly correct in the parts they assign to different foods, is too large a question for discussion here. This is a point upon which it is probable men of science will agree to differ.

*Maid Marian* should ask a medical man to examine her heart, &c., and let him decide what anæsthetic would be most suitable for her.

*Male Attendant* should consult a surgeon. It is quite probable that the case of knock-knees he inquires about is curable.

152 *Stewardess (E. H.).*—See answers to queries in The Hospital of September 8th.

### Wants and Workers.

A lady wishes to find a comfortable home in the country for a farmer's wife, aged 65, who had a slight attack of paralysis in the summer, and now suffers from loss of memory. She is excitable and restless. Write, stating terms, to C. C.

### Nursing in Holland.

By a Dutch Correspondent.

As a direct result of the course of instruction given by Dr. C. B. Telanus, a branch of the Society for Rendering First Aid to the Injured has been established in Amsterdam, and seems likely to flourish there. At the first meeting sixty persons expressed their willingness to become members, while twenty others had previously joined the society. The Queen and the Queen-Regent have each sent a donation of a hundred francs as a token of their interest in the work.

The new hospital of St. Elizabeth at Arnhem has been opened with all due ceremony, a choral service being held on the occasion. The building is a very imposing one. That part of it which is already completed (it is proposed at some future date to add another wing) contains, on the ground floor, the kitchens, heating apparatus, and dining-rooms for nurses and servants; on the first floor, a large hall, waiting-rooms, doctors' rooms, operating-room, &c.; and on the second floor are the wards.

The Association for the Christian Care of the Insane in the Netherlands held its tenth annual meeting a short time ago in the Hall of Arts and Sciences at Utrecht, Herr L. Lindeborm, of Kampen, presiding. The reports of the progress of the society were decidedly favourable; it has now established forty-five branches, with a total of 5,030 members. Permission has been obtained to erect at Zuidlaren an asylum with accommodation for 350 patients—162 men and 188 women.

The Eye Hospital at Utrecht has received substantial assistance from the picture exhibition and lottery held a short time ago for its benefit. The institution is the richer for the undertaking by the sum of 15,200 francs.

At the Wilhelmina Hospital, Amsterdam, Brother Poppée, the senior male nurse, was, the other day, the central figure of a little ceremony of congratulation. Brother Poppée has been in the service of the institution for more than twelve years—first at the old Buiten Hospital, and recently in the new Wilhelmina Hospital. After receiving from the head of the institution hearty congratulations, Brother Poppée was the recipient of numerous proofs of the esteem in which he is held by his fellow workers.

The second annual report of the Protestant Deaconesses' Institution, at Vlaardingen, has been issued. The work of the sisters among the poor continues to extend, and especial mention is made of Sister Gretha Hofstra for her zeal and energy during the past year. From July 1st, 1893, to July 1st, 1894, she paid no fewer than 5,322 visits to her patients. The help of the sisters was required during the cholera "season," at which time they had charge of the two cases—one of them fatal—which appeared in the town. The surplus of the institution forms a fund out of which pensions are granted to sisters who are forced by advancing age to relinquish their work.

The *Dutch Journal of Medicine* announces that Amsterdam contributed to the Buda-Pesth Congress a collection of charts and models illustrating the present system of public sanitation.

The central committee of the "Red Cross" has sent the sum of 4,000 francs to their committee in the Dutch East Indies as a contribution to the costs of the assistance rendered to the troops on their expedition to Lombok. This donation is not sent in consequence of the unfavourable news from Lombok, but is a result of the representations of the Indian committee, which has already sent 8,000 francs to Lombok. The biennial meeting of the society takes place this month at The Hague. It is proposed, in future, that after eight years of unbroken work the nurses of this association shall receive 400 francs; also that pensions shall be given, on the completion of twenty years' service, to those who are over fifty years of age. At Beetsterzwaag (Friesland) a hospital for the reception of the sick poor is being established by Jonkvrouw De Vos van Steenwijk. At first only children will be received; but, if the institution succeeds, other persons will be admitted.

### The Book World for Women and Nurses.

[We invite Correspondence, Criticism, Enquiries, and Notes on Books likely to interest Women and Nurses. Address, Editor, The Hospital (Nurses' Book World), 428, Strand, W.C.]

Nursing: Its Principles and Practice. For Hospital and Private Use. By Isabel Adams Hampton, Graduate of the New York Training School for Nurses attached to the Bellevue Hospital; Superintendent of Nurses and Principal of the Training School for Nurses, Johns Hopkins Hospital, Baltimore, Ind.; late Superintendent of Nurses, Illinois Training School for Nurses, Chicago, Illinois. Illustrated. Second Edition. London: The Scientific Press (Limited), 428, Strand, W.C. 1894.

Little more than a year ago we noticed Miss Hampton's text-book on nursing (August 12th, 1893, p. cxcviii.) That the second edition, which we have now before us, should have been called for so soon is in itself a strong testimonial to the value of the work. It is thoroughly practical, and the chapters devoted to the principles and methods of training nurses give it a special value and interest for matrons and superintendents. Miss Hampton's regret that there is no post-graduate course for nurses who have completed a two years' curriculum will be echoed by all those who have at heart a high standard for the nursing profession; but, perhaps, especially as post-graduate courses can never be compulsory, the end aimed at will be best attained by the training schools extending their course to three years. As the nurse in her third year is the most useful to the hospital, the school would have no reason to regret the prolongation. At the same time we think that Miss Hampton's scheme of training for a two years' course is altogether admirable; but as the whole twenty-four months are occupied by it, it could not, as a matter of practice, be carried out in its entirety, as it leaves no time for vacation or night duty, which, therefore, could only be found by shortening the training in the medical, surgical, and gynæological wards. The month devoted to the cooking school is, on the other hand, a distinct advantage to the suggested training; and it would be well if no private or district nurse were considered fully qualified without a certificate in sick-room cookery.

Miss Hampton's chapter on the equipment of a ward is specially to be commended to the attention of managers, who are sometimes inclined to economise in this matter. The suggested patients' toilet-basket for each nurse would certainly be a great aid to promptitude in preparing patients for the day, and would probably save much irritation. But, unfortunately, it is just one of the things which authorities would incline to consider unnecessary. On the other hand, they would endorse most readily Miss Hampton's wise remarks to nurses on the subject of hospital economy. With these (*i.e.*, hospital supplies) the nurses have more to do than anyone else; they are the stewards, and upon them lies the responsibility of seeing that nothing is wasted or used extravagantly. For instance, the laundry work could often be greatly lessened if nurses were more careful when changing beds and using towels; and the gas bill could be much reduced if they would remember to turn down or put out the gas when it is not needed, or when half a flame would do instead of a full one; alcohol, drugs, milk, and food should be ordered only in such amounts as are necessary, and one should be economical, although not parsimonious, in their use. Two bandages should never be put on when one would answer. In all these ways the nurse may become a liberal contributor to charity in that through her efforts two patients can be cared for where otherwise only one could be supported."

That the nursing instructions given are admirable need hardly be said, and they are given in a clear, concise, and practical style, which will impress them on the memory of the reader. We think, however, that in the chapter on obstetric nursing the importance of the vaginal douche even in ordinary cases is not sufficiently dwelt on. Even when there is no threatening of hæmorrhage it adds greatly to the patient's comfort, and mitigates the offensiveness of the later *lochia*. Miss Hampton is also not at one with English authorities in advising that after the first bath a new-born child should not receive another until the cord has fallen off. These are the only points we have noticed in which the book may be regarded as old-fashioned. Miss Hampton gives very clear directions as to the performance of those minor operations which in an emergency it may fall upon the nurse to perform, while at the same time she carefully discourages the conceited tendency not unknown among some to undertake surgical work on their own responsibility. In the same way, in her careful and detailed advice as to the observation of symptoms, on which the doctor has to rely on the nurse, she points out that it is not for the nurse to judge what are important and what are trivial, but that all should be noted and narrated. Indeed, the tone and temper of the book as much as its technical instructions commend it for the use of both student and experienced nurses.

A word is due to the appearance of the book, which is now issued by the Scientific Press (Limited). The size is convenient, the print and paper admirable, and the illustrations remarkable for clearness. Both form and contents render this volume an admirable nurses' companion.

### A Case for Assistance.

We have received a most grateful letter from Mrs. E. E. Mortimer, the old matron in whose case our readers have taken so great an interest, and towards whose support they have subscribed £31 0s. 6d. Mrs. Mortimer states that she must have given up her shop this winter if it had not been for the help afforded her. For several years past she has struggled on, battling with the increasing infirmity of years, and hampered by an old debt caused by the dishonesty of a person whom she trusted. Her household consists of a dependant aged sixty, and an orphan child of twelve. The £30 subscribed has enabled her to clear off all debts, and to increase her stock of goods, leaving a small sum in hand for contingencies. As the shop brings in sufficient each week to enable her to pay her rent and to maintain her household comfortably, it will be seen that the generosity of our readers has provided the deserving ex-matron with all necessary comforts, and secured for her a fair prospect of freedom from worry for the rest of her life. In again congratulating those who have subscribed so generously, we feel that our thanks and theirs are due to Miss Bell and Miss Farrow, who first took up the case, and who have been assiduous in their management of the correspondence, which must have entailed considerable trouble upon one or both of them.

### Appointments.

Birmingham and Midland Homœopathic Hospital.—Miss Tamar E. Bean has been made Lady Superintendent of this hospital. She was trained for three years at the Hospital for Sick Children, Great Ormond Street, and was Charge Nurse at the East Suffolk Hospital, Ipswich, for two years; Ward Sister at Birmingham Infirmary for three years, and Night Superintendent and Home Sister at the same institution. Miss Bean takes many good wishes to her new work.

Chorlton Workhouse Hospital.—Miss Mary Rawson has been appointed Matron of the Chorlton Workhouse Hospital, Withington, Manchester. Miss Rawson was trained at the Brownlow Hill Infirmary, Liverpool, and for the last five years has worked at the Infirmary, Birmingham. She has held the post of Sister in both medical and surgical wards, and for two years filled the responsible position of Night Superintendent. We congratulate Miss Rawson on her appointment, and wish her every success.

Victoria Infirmary, Glasgow.—Miss. M. M. McFarlane has been appointed Matron of this hospital. She was trained at the Nightingale School, Liverpool, and was for two years Head Surgical Nurse at Burton-on-Trent Infirmay, and Matron for one year at the Cottage Hospital, Burton-under-Reedwood, five and a half years at Bothwell Asylum, four years at Woodilee Asylum, and for the last four years Matron and Lady Superintendent at Barnhill Hospital, Glasgow. We congratulate Miss McFarlane on an appointment which her large experience specially fits her for, and we wish her every success.

